# Supplementary material for: Ultrastructural and Functional Characterization of Mitochondrial Dynamics Induced by Human Respiratory Syncytial Virus Infection in HEp-2 Cells
Source: Viruses. 2023 Jul 7;15(7):1518. doi: 10.3390/v15071518 (PMC10386036; doi:10.3390/v15071518)
Supplement: Supplementary file 1 [file viruses-15-01518-s001.zip › viruses-2454150-supplementary.pdf]

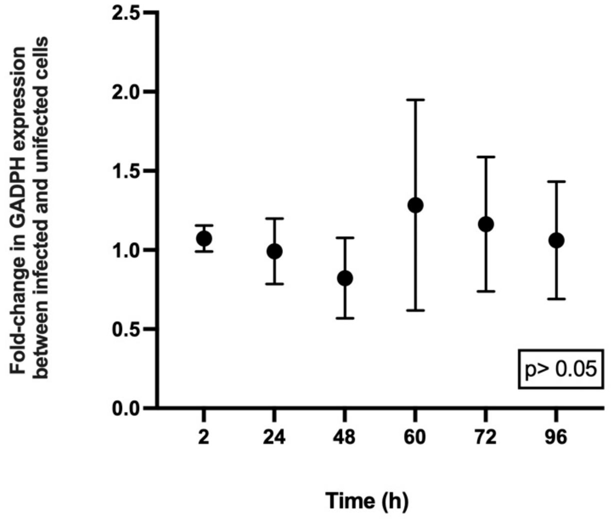

Figure S1. Fold-change in GADPH expression between infected and uninfected cells. The fold change in gene expression was calculated using the 2-ΔCt method. The values represent mean fold change with standard deviation. No statistically significant relationship exists between infected and uninfected cell expression of GAPDH.

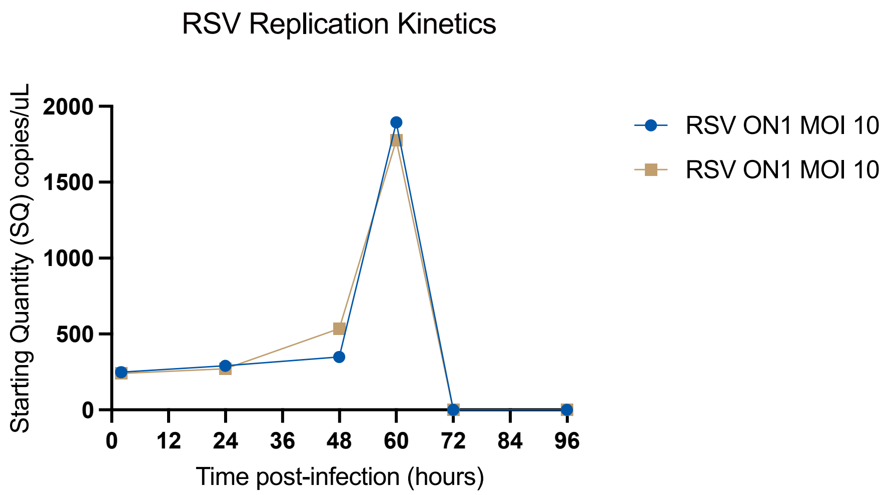

Figure S2. Kinetics of hRSV ON1 infection in vitro assays. Hep-2 cell monolayers were infected with hRSV ON1 at a multiplicity of infection (MOI) 10. At various times post-infection, total virus (copy/uL) in the total RNA were determined. In each assay, the experimental data (circles) were collected in duplicate (MOI 10). The effect of hRSV infection at 60-h.p.i. was analyzed only by RT-qPCR but not for the ultra-structural analysis.
